# Inferring Gene Networks for Strains of *Dehalococcoides* Highlights Conserved Relationships between Genes Encoding Core Catabolic and Cell-Wall Structural Proteins

**DOI:** 10.1371/journal.pone.0166234

**Published:** 2016-11-09

**Authors:** Cresten B. Mansfeldt, Gretchen W. Heavner, Annette R. Rowe, Boris Hayete, Bruce W. Church, Ruth E. Richardson

**Affiliations:** 1 Department of Civil and Environmental Engineering, Cornell University, Ithaca, NY, United States of America; 2 Field of Microbiology, Cornell University, Ithaca, NY, United States of America; 3 GNS Healthcare. Cambridge, MA, United States of America; Naval Research Laboratory, UNITED STATES

## Abstract

The interpretation of high-throughput gene expression data for non-model microorganisms remains obscured because of the high fraction of hypothetical genes and the limited number of methods for the robust inference of gene networks. Therefore, to elucidate gene-gene and gene-condition linkages in the bioremediation-important genus *Dehalococcoides*, we applied a Bayesian inference strategy called Reverse Engineering/Forward Simulation (REFS^™^) on transcriptomic data collected from two organohalide-respiring communities containing different *Dehalococcoides mccartyi* strains: the Cornell University mixed community D2 and the commercially available KB-1^®^ bioaugmentation culture. In total, 49 and 24 microarray datasets were included in the REFS^™^ analysis to generate an ensemble of 1,000 networks for the *Dehalococcoides* population in the Cornell D2 and KB-1^®^ culture, respectively. Considering only linkages that appeared in the consensus network for each culture (exceeding the determined frequency cutoff of ≥ 60%), the resulting Cornell D2 and KB-1^®^ consensus networks maintained 1,105 nodes (genes or conditions) with 974 edges and 1,714 nodes with 1,455 edges, respectively. These consensus networks captured multiple strong and biologically informative relationships. One of the main highlighted relationships shared between these two cultures was a direct edge between the transcript encoding for the major reductive dehalogenase (*tceA* (D2) or *vcrA* (KB-1^®^)) and the transcript for the putative S-layer cell wall protein (DET1407 (D2) or KB1_1396 (KB-1^®^)). Additionally, transcripts for two key oxidoreductases (a [Ni Fe] hydrogenase, Hup, and a protein with similarity to a formate dehydrogenase, “Fdh”) were strongly linked, generalizing a strong relationship noted previously for *Dehalococcoides mccartyi* strain 195 to multiple strains of *Dehalococcoides*. Notably, the pangenome array utilized when monitoring the KB-1^®^ culture was capable of resolving signals from multiple strains, and the network inference engine was able to reconstruct gene networks in the distinct strain populations.

## Introduction

Organohalide-respiring communities of microorganisms have been utilized at field sites to bioremediate common chlorinated organic pollutants [1, 2]. These pollutants include pervasive industrial solvents of the chlorinated ethene class (tetrachloroethene (PCE), trichloroethene (TCE), dichloroethene (DCE), and vinyl chloride (VC)). In these communities, the organisms that preform the crucial step of reductively dechlorinating completely to ethene (ETH) are strains of *Dehalococcoides mccartyi* (*Dhc*; [3]). *Dhc* is an obligate hydrogenotrophic dehalorespiring anaerobe, requiring hydrogen as an electron donor and halogenated organics as an electron acceptor. The varied strains of *Dhc* each contain a strain-specific suite of multiple reductive dehalogenases (RDases). RDases are the enzymes responsible for the replacement of a halogen with a hydrogen, thereby reducing the carbon atom [4, 5]. Each strain appears to harbor a unique suite of RDases and expresses a subset of these RDases in response to halogenated substrates [6, 7].

Although the general metabolic characteristics of *Dhc* are described, multiple questions remain as to the relationships among metabolic and regulatory proteins within the cell including the mechanism for energy generation. Previously, central metabolic genes such as the citrate synthase [8] and genes encoding for hypothetical proteins up-regulated during starvation [9] were heterologously expressed in a model organism. Additionally, a method to heterologously express RDases was demonstrated for the VC-reductase, VcrA [10], enabling further biochemical characterization of *Dhc* strains. However, such biochemical approaches are too costly and time-consuming to apply to all genes encoded in a genome. Additionally, *Dhc* remains a genetically intractable organism, precluding knockout and *in vivo* over-expression studies [11]. Therefore, the function of and relationships between these proteins of interest must be described through alternative methods.

An approach to predict the metabolic and regulatory networks of an organism is to rely on statistical inferences of high-throughput data from genome wide expression studies [12]. Previous investigations have utilized various techniques, such as differential expression analyses and gene clustering, to relate transcript abundance to an experimental condition and to predict the function of the enzymes encoded on the expressed transcripts in *Dhc*. These studies include investigations of *Dhc* as the organism transitions from exponential growth to stationary phase [13], biosynthesizes molecules through the central carbon metabolism pathway [14], acquires and modifies key cobalamin cofactors required for growth [15], fixes nitrogen [16], or grows under nitrogen limitation [17]. These transcriptomic and proteomic studies have shown success in producing, testing, and supporting hypotheses.

To interpret the data resulting from these high-throughput studies, computational tools such as network analyses are available. Investigations targeting *Dhc* utilized these methods to begin to hypothesize roles for the genes that lacked a predicted function (nearly one-third of the approximately 1,600 genes on the *Dhc* genome). Two previous studies applied network analyses to recover gene network topology from high-throughput transcriptomic data. A transcriptomics-based clustering analysis previously identified functionally enriched clusters for *Dhc* to predict the function of poorly annotated genes that were subsequently experimentally validated [18, 19]. Additionally, a Bayesian analysis based on clusters of gene expression profiles was conducted for the *Dhc* strain 195 population in D2 to link these clusters to the investigated experimental conditions, uncovering novel stress related relationships [20]. However, by relying on clustering prior to network inference, both of these studies were unable to detect nuanced gene expression relationships.

Additional computational tools that employ data driven hypotheses of fine-scale gene network topologies can infer the experimental drivers of gene expression [21]. In this study, a large-scale Bayesian network inference analysis entitled Reverse Engineering/Forward Simulation^™^ (REFS^™^; GNS Healthcare, Cambridge, MA) was applied to microarray datasets to infer robust relationships among the larger number of individual variables in the datasets [22]. The nodes, which are the random variables sampled (in this investigation the microarray gene transcript intensities, metabolite data, and experimental conditions) are linked through edges, which capture the relationships that are established between two or more variables based on the analyzed dataset [22]. Because either an experimental condition or a gene expression profile can serve as a node, the model is able to uncover strong gene-gene or gene-condition relationships for *Dhc*, assisting in the description of likely pathways and the prediction of the role of proteins with unknown function. Gene-gene relationships may identify known protein complexes that interact in novel ways to produce the phenotypic characteristics of *Dhc*. Additionally, gene-gene relationships can identify linkages between enzymes with known or well-predicted functions with those with minimal or no annotation, developing hypotheses for the role of proteins with unknown function. Gene-condition relationships provide stronger evidence for predicting the performance of these proteins with unknown function; directly linking the expression of a transcript with an experimental condition (e.g., a stress or growth limitation) provides a succinct hypothesis of the cellular role of the encoded protein. In the employed Bayesian framework, these gene-condition relationships are more easily determined when the dataset covers a diverse set of experimental perturbations. Therefore, the considered datasets were designed to analyze multiple stress and growth conditions to ensure a broad and diverse set of experimental interventions.

To determine the transcriptional relationships unique to and shared across strains of the non-model *Dhc*, we employed a REFS^™^ analysis directly on mixed-type datasets to determine the gene expression networks for two distinct mixed-community, organohalorespiring cultures: D2 (Cornell University; Ithaca, NY) and the commercially available KB-1^®^ (SiREM Laboratories; Guelph, Ontario Canada). D2 was selected for this investigation because this culture is well studied and contains one *Dhc* population (*Dhc* strain 195) [20, 23]. The KB-1^®^ culture is used commercially for bioaugmentation and contains multiple *Dhc* populations and a dechlorinating *Geobacter lovleyi* strain [24]. Both cultures have publicly available metagenomes, enabling the application of microarrays (a strain 195 specific microarray for D2; a pangenome microarray for KB-1^®^) to monitor the transcriptome of the *Dhc* populations. Additionally, both cultures were subjected to a sampling campaign that was comprised of multiple perturbations including the application of stress conditions. Therefore, from the separate consensus network reconstructions performed on the transcriptomic and metabolite data for the D2 and KB-1^®^ cultures, we anticipated identifying linkages between these diverse experimental conditions (such as the type of substrates applied to cultures and the rates being fed) and gene transcripts. We then compared these two consensus networks to elucidate the conserved gene networks among *Dhc* species (with a focus on transcripts encoding RDases and respiration-linked enzymes) and strain specific behavior. Combined, the recovered networks can highlight the strongest gene-gene and gene-condition relationships in the environmentally important *Dhc* genus and provide insights into the mechanisms by which these organisms respire organohalides.

## Materials and Methods

### Culture growth

The Donna II (D2) liquid culture maintained at Cornell University under conditions previously described [6] and the commercially grown KB-1^®^ (SiREM; Guelph, Canada) were the starter cultures for the D2- and KB-1^®^-based experiments, respectively. Each culture bottle contained 100 mL of culture and 60 mL of 70/30% N_2_/CO_2_ (AIRGas, Radnor, PA) headspace. The KB-1^®^ commercial culture was provided by SiREM Laboratories (Guelph, Ontario). After receipt, the liquid KB-1^®^ was stored at 4 C under anaerobic conditions for less than one week and was acclimated to room temperature for approximately 12 hours prior to use.

### D2 experimental conditions

The D2 experimental conditions have been summarized in previous publications [20, 25, 26]. In brief, the dataset used consists of complimentary DNA (cDNA) microarray and metabolite data from continuously-fed experiments. The cultures in these experiments were delivered varied rates and types of electron donor (butyrate (60% w/v; ACROS Organics, Geel, Belgium), lactate (99+%; ACROS Organics), yeast extract (bacteriological grade, MoBio, Carlsbad, CA), fermented yeast extract, and no donor) and acceptors (PCE (99+%, Sigma Aldrich, St. Louis, MO), TCE (99+%, Sigma Aldrich), cis-DCE (99+%, Sigma Aldrich), 2,3-dichlorophenol (DCP; 99+%, ACROS Organics), and no electron acceptor). Electron acceptor feed rates varied from 0 to 481 microeeqL^−1^ h^−1^. As detailed in Mansfeldt et al. [26], electron equivalents (eeqs) are the number of moles of electrons respired during the process of organohalorespiration. In these experiments the D2 culture respires six, four, two, and two eeqs in the reduction of PCE, TCE, cDCE, and DCP, respectively (the KB-1^®^ culture respires eight, six, four, and two eeqs in the reduction of PCE, TCE, cDCE, and VC, respectively). Electron donor-to-acceptor ratios varied from 0 to 17 in terms of overall microeeq. In total, 47 continuously fed D2 experiments were considered in the final analysis. A full summary of experimental conditions utilized in the analysis is detailed in S1 Table.

### KB-1^®^ experimental conditions

The experimental conditions run on the KB-1^®^ culture were, in brief, a batch-fed time-series (13 100-mL subcultures of KB-1^®^; a time-zero sample and duplicate samples were sacrificed at 4.2, 8.3, 13.7, 23.1, 27.9, and 69.7 hours post batch feeding of TCE (99.5%, Thermo Fisher Scientific, Waltham, MA), hydrogen (AIRGas, Radnor, PA), and acetate (99.7%, Thermo Fisher Scientific) as previously described [27] (S1 Fig)), a batch oxygen stress experiment (seven samples; one time-zero sample, two with no stress, two sampled six hours after stress, two sampled 48 hours after stress), a batch-fed 1,1,1-trichloroethane (TCA; 99+%, ACROS Organics) stress experiment (seven samples; one time-zero sample, two with no stress, two sampled six hours after stress, two sampled 48 hours after stress (S2 Fig)), and a continuously fed TCE steady-state experiment (seven samples; one control, three sets of duplicates fed TCE at 2.7, 6.8, and 16.5 μmol L^−1^ h^−1^[27]).

### Metabolite monitoring

Flame ionization detection (FID) gas chromatography (GC) was used to determine the chlorinated ethenes, ethene, and methane concentration levels using previously described methods [28]. High concentrations of methane and hydrogen were analyzed by a GC equipped with a thermal conductivity detector (TCD). Low hydrogen levels were measured by a GC with a reduced gas detector (RGD; Trace Analytical, Muskegon, MI; SUPELCO 100/120 Carbosieve G 10’x1/8”ï¿½ column, Sigma Aldrich). Chlorophenols were extracted and assayed with a GC-FID as previously described [26]. In addition to gaseous components, the levels of acetate and butyrate in the D2 mixed cultures were monitored by ion chromatography (Dionex AS50, Dionex, Sunnyvale, CA; IONPAC AS14A 4x250mm column) as previously described [27].

### Nucleic acid extraction and analysis

The microarray protocol followed in this study was previously described [26]. In brief, from each of the biological duplicates or triplicates, 50 mL of mixed culture was centrifuged for 10 minutes, the supernatant was decanted, and the cell pellet was frozen at -80 C for under one week. RNA was extracted and purified from the cell pellet by a modified RNEasy (Life Technologies, Grand Island, NY) protocol [23, 29], and the resulting RNA was frozen at -80 C for under one week. Contaminating DNA was then removed by DNAse digestion (TurboDNAse; Life Technologies). The purified RNA was reverse transcribed (Superscript II; Life Technologies) with an amino-allyl dUTP (GE Healthcare, Little Chalfont, UK) into complimentary DNA (cDNA). The cDNA was then frozen at -20 C until processing for the microarray. After purification (base hydrolysis and column cleanup (CyScribe DNA Columns, GE Healthcare)), the cDNA was then labeled by staining with Cyanine-3 or -5 (Cy3 or Cy5, GE Healthcare). Either 400 ng of D2 or 800 ng of KB-1^®^ from the labeled cDNA pool was then washed and hybridized by the Cornell University Core Life Sciences Division to an Agilent Two-Color 8-plex 15 K [26] or 4-plex 30 K [30] microarray, respectively, as described below.

### Microarray platform description

The D2 microarray (GEO platform GPL11218) was designed specifically for the genome of *Dhc* strain 195, the only *Dhc* strain in the D2 community (confirmed by a metagenome analysis of D2 which is freely available at IMG MER). A single 60-mer probe was designed for each gene of interest using the Agilent eArray software suite (Agilent Technologies, Carlsbad, CA, USA), as previously described [26]. The final array design consisted of probes for genes on the genome of *Dhc* strain 195, 16S rRNA sequences for D2 microbial community members, several functional genes from non-*Dhc* community members (hydrogenases), and a luciferase internal standard. The design of the microarray ensured specificity for the *Dhc* signal by selecting probes that did not display crosstalk with other community members found in the publicly available metagenomes (including metagenomes for both cultures studied here) or with other organisms outside of the *Dhc* genus in the NCBI database. Each probe (n = 1,628) was replicated at least eight times in spots distributed across the array.

The pangenome microarray used to analyze the commercial KB-1^®^ culture has been described and validated for use with mixed cultures previously [30]. This pangenome array was constructed to target each gene in *Dhc* strains with a publicly available genome at the time of design (*Dhc* strains 195, BAV1, CBDB1, GT, and VS) and the *Dhc* strain recovered from the metagenome of the laboratory-maintained KB-1 culture [31, 32]. Therefore, the design contained multiple probes per orthologous gene cluster. The probes have lengths between 49 and 60 nucleotides. Each probe (n = 7,506) was replicated in at least four spots distributed locations across the array. The probes designated with the RC nomenclature throughout the text were designed to target a different region of the coding strand. This region was selected by considering the reverse compliment sequence and then transcribing the final designed probe.

### Microarray data processing and normalization

The microarray data from both the arrays following cDNA hybridization were obtained from an Agilent Technologies Scanner G2505C using Agilent’s Feature Extraction Software (Agilent Technologies). The data was background corrected and LOESS normalized. Intensities from identical probe spots were geometrically averaged. The raw data from the D2 and pangenome microarray are freely available at the NCBI GEO database under accession numbers GSE26288 and GSE42136, respectively.

For both the D2 and pangenome microarrays, spots that did not display an intensity value above at least two standard deviations greater than the background fluorescence intensity in at least one sample were removed from the dataset. This filter selects for gene expression values that were confidently detected above background. The list of probes exceeding this cutoff on the pangenome array includes multiple sequences targeting the orthologous gene from different strains (higher than 85% identity along the length of the probe), often with overlapping targeted sequences. Therefore, the pangenome probe-set was sorted to bin probes that target a conserved orthologous gene across multiple *Dhc* strains. When the expression was highly correlated (r > 0.9) for probes designed for the orthologous gene, the data from the probe displaying the higher intensity was used in the final dataset. For example, when three probes target an orthologous gene and only two probes display r > 0.9, the higher intensity probe for the correlated probes and the distinct probe would both be represented in the final dataset. The microarray dataset for the D2 array run sampling the D2 culture and the pangenome array sampling the KB-1^®^ culture consisted of 47 (previously described; [26]) and 24 samples, respectively.

### Reverse Engineering/Forward Simulation^™^ network modeling

The network reconstruction process utilized the Reverse Engineering/Forward Simulation^™^ (REFS^™^) analysis designed by GNS Healthcare and was run in the R environment (R version 3.0.2). This Bayesian reconstruction considers all of the continuous variables (the ratio of the red to green channel for the transcripts monitored on the arrays, the chemical concentrations of metabolites and inhibitors, the organohalorespiration rates, and the experimental setup parameters) as nodes in the network with acyclic directed relationships represented as the edges, as previously described [33]. Zero values were not allowed within the metabolite data; therefore, when a metabolite or experimental parameter was below detection or zero, either the detection limit or a value of 10^-4^ was used. In these cases, a censoring switch was set to 0 (the ‘censored’ state). Additionally, the entire dataset was eighth root transformed prior to REFS^™^-based inference.

In addition to the continuous variables within the network, discrete variables (e.g., binary operators describing the state of the culture, including censoring switches, and tiered operators partitioning the dataset for the electron acceptor type) were allowed to be modulators of node-node relationships. These modulated relationships in the network were considered to be conditional relationships and could take the following forms: linearly-conditional (edges that are present only with a discrete variable pattern during specific growth conditions; e.g., only when cDCE fed) and switched-conditional (edges that are present when a binary discrete variable (e.g., a censoring switch is set to one). The full spreadsheet of the considered discrete and continuous experimental variables and censoring switches for the D2 model are presented in S1 Table.

**Fig 1:**
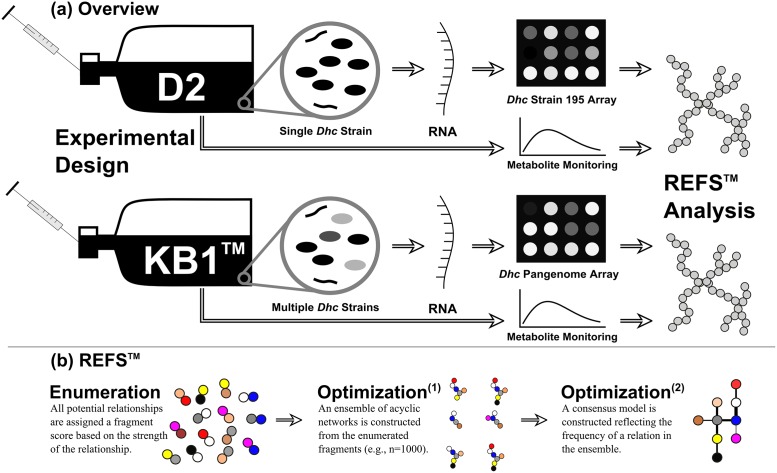
Diagram of the experimental and analytical procedures. (a) Overview of the experimental design. The transcriptome of the continuously or batch fed D2 (single strain) or KB-1^®^ cultures were monitored using microarrays, and the metabolite data was collected through chromatographic techniques. (b) Summary of the REFS^™^ gene network inference process. All possible edges are assigned a score based on the data in the enumeration step. Optimized networks and a consensus network are constructed from these enumerated fragments.

The REFS^™^ network reconstruction was run in two steps: enumeration of small network fragments and optimization of the final network ensemble. The overall work flow in this study to reconstruct the final REFS^™^ network is presented in Fig 1. The enumeration step considered all possible relationships (the fragments) in the network and scored the likelihood of the fragment given the data. The optimization step then sampled these relationships (the fragments) to assemble an ensemble of 1,000 networks to develop the final network. The strength of the relationship was then determined by a frequency score (f). This f displays the fractional value of the number of times a specified relationship was retained in the set of 1,000 constructed networks in the optimization step. For example, when f = 0.6 for a specified relationship, the represented relationship appears in 600 of the 1,000 assembled networks. The details of these enumeration and optimization steps have been previously presented [21, 33].

## Results and Discussion

### Microarray data reduction and strain differentiation in the KB-1^®^ community

**Fig 2:**
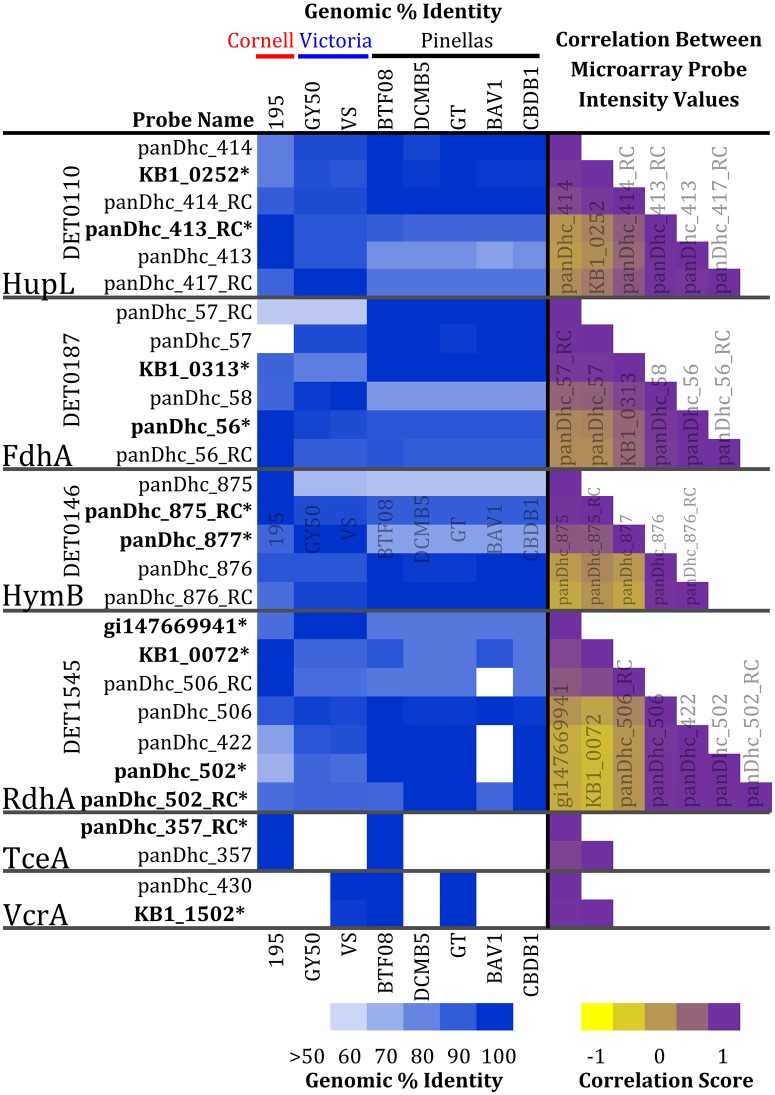
Ordering *Dhc* pangenome array probes based on sequence similarity and captured expression profiles for the KB-1^®^ culture. The array contains multiple probes for *Dhc* orthologs. The white-to-blue shaded columns (left) display the genomic % identity of the probe sequence to gene sequences for representative members of the Cornell, Victoria, and Pinellas groups of *Dhc*. The yellow-to-purple columns (right) represent the correlation relationship scores of the probe intensity across all cDNA pools from all samples. Bolded (*) probes indicate those that were retained for the REFS^™^ analysis of the KB-1^®^ data.

The KB-1^®^ dataset required initial preprocessing steps to remove redundant probes from the analysis (to increase the sensitivity) using a correlation filter. In total, the pangenome array was designed with 7,506 different probes [30]. Because of the design of the pangenome array, the array potentially can discern multiple strains of *Dhc* within a single community, but the array also has a degree of redundancy because multiple probes can target the identical transcript. KB-1^®^ is known to contain multiple strains of *Dhc* [24]. To minimize this redundancy, the data reduction process employed two steps. First, the spots on the microarray that did not report an intensity that exceeded two standard deviations above the background intensity value in at least one experiment were not considered. Second, probes that were designed for othologs across *Dhc* strains were compared to identify and remove strongly correlated probes; probes with correlations scores exceeding 0.9 were collapsed into a single probe intensity value by utilizing the probe that displayed a higher average intensity on the array. An example of this comparison for selected probes is shown (Fig 2). The heatmap in blue presents the genomic identity match of the probe to the indicated strain, and the heatmap ranging from yellow to purple visualizes the pangenome microarray correlation score between the probes. For example, six probes designed for the conserved [Ni Fe] hydrogenase, HupL, were present on the array, targeting strains in the Cornell or Victoria (three probes) and Pinellas (three probes) phylogenetic groups (the probe-target percent identity shaded in red in Fig 2). When investigating the correlations between levels of these six transcripts, distinct and strong correlations were noted, displaying two distinct groups in transcript expression that corresponded to two groupings of *Dhc* strains (i.e., the Cornell with Victoria group and the Pinellas group). Therefore, only the intensity values from two probes targeting the HupL transcripts KB1_0252 and panDhc_413_RC were used as representatives of the Cornell/Victoria and Pinellas groups, respectively, in the network analysis (Fig 2, bolded probes). When this correlation and genomic-similarity filter process was applied to all probes on the array, the data from the 7,506 pangenome probes collapsed into 2,627 unique gene expression patterns (the probes remaining are detailed in S2 File). This data reduction process eliminates pangenome redundancy without losing the ability to monitor multiple *Dhc* strains in KB-1^®^.

To determine whether the pangenome array distinguished more than one strain in the KB-1^®^ community, the expression profiles of several key oxidoreductases and other enzymes are highlighted in Fig 2. Notably, probes for conserved orthologous genes within the *Dhc* genus appear to be specific for one *Dhc* group over the other, as expected from the design of the array. This preferential targeting is also seen in the correllogram for the expression captured on the array. The correllogram displays the correlation score between the data for all probes on the array for an orthologous transcript. Probes that target the Cornell/Victoria clade display a higher positive correlation (shaded purple) with intensity data for other probes targeting the identical clade but a lower correlation (shaded yellow) to intensity data from probes that target the Pinellas clade (and vice-versa).

### Network sizes and structures

**Fig 3:**
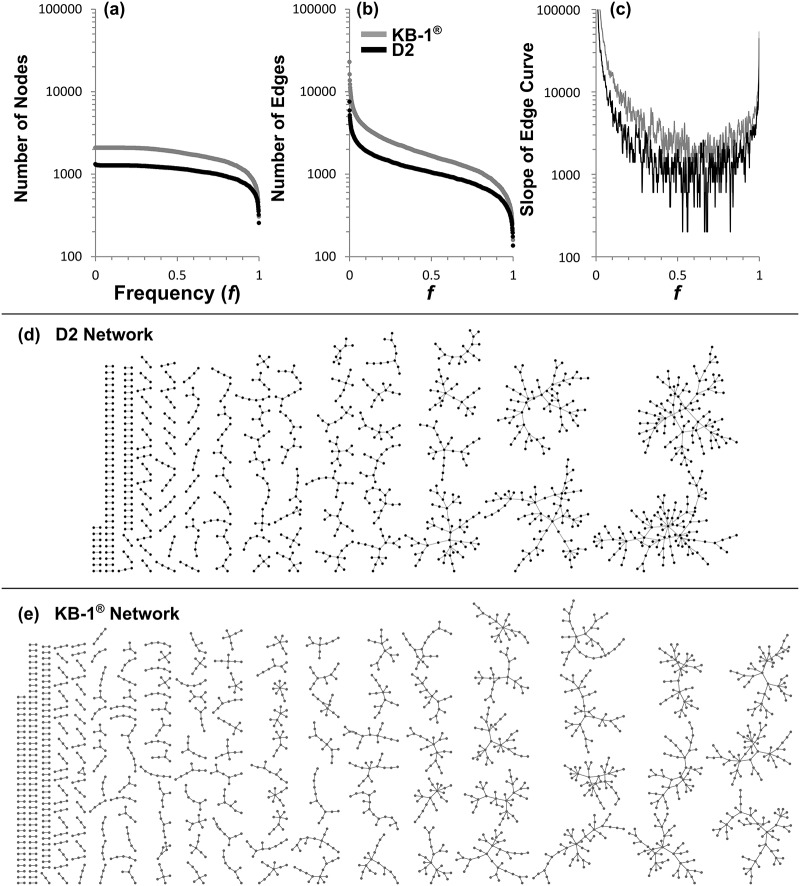
REFS^™^ network summaries for the D2 (black) and KB-1^®^ (grey) networks. The number of (a) nodes and (b) edges remaining in the network is plotted against the frequency cutoff established for the edges. (c) The slope of (b) versus frequency; the minimum was observed at approximately f = 0.6 for both networks. (d) The D2 consensus network of all edges with f ≥ 0.6. (e) The KB-1^®^ consensus network of all edges with f ≥ 0.6. More detailed (e.g., with labels and edge strengths) consensus networks are available in S3 and S4 Files.

The KB-1^®^ REFS^™^ analysis considered the data from 2,627 probes. In comparison, data from 1,488 probes on the *Dhc* strain 195 specific microarray were used in the REFS^™^ analysis of the D2 community. The data files used to construct the KB-1^®^ and the D2 consensus networks are provided as supplementals (S1 and S2 Files, respectively). The number of nodes (Fig 3a) and edges Fig 3b) in the consensus network is plotted versus the edge frequency cutoff, and the significant frequency (f) cutoff is also identified for both models as f ≥ 0.6 (Fig 3c). Taking all identified connections into account (f ≥ 0.001), the D2 model had 1,322 nodes with 7,553 edges, and the KB-1^®^ network had 2,087 nodes with 22,974 edges. The significance cutoff for these networks (establishing the consensus network) was set for the point at which the slope of the edge curve displays a minimum—this set point allowed a qualitative selection of a broad set of edges enriched for true positives, since false positives would be expected to occur at low frequencies and in high numbers on the left side of the cumulative distribution of edge frequencies; this value was found to be approximately f = 0.6 for both datasets (Fig 3c), and therefore an edge had to appear in 600 out of the 1,000 networks constructed to be retained in the final consensus network. After applying this constraint, the D2 consensus network had 1,106 nodes with 794 edges, and the KB-1^®^ consensus network had 1,715 nodes with 1,458 edges. The details of the consensus networks for the D2 (Fig 3d) and KB-1^®^ (Fig 3e) cultures are available as a network file (S3 and S4 Files, respectively). Both of these consensus networks were sparse with only 0.72 and 0.85 edges per node for the D2 and KB-1^®^ datasets, respectively.

### Edges driven by experimental conditions

The datasets were used to discover novel relationships between transcripts and experimental conditions. In the D2 consensus network, the experimental conditions were primarily connected to other experimental conditions, displaying few connections to gene transcripts. Although continuous variables associated with culture conditions tended to self-associate in the consensus network, several discrete variables influenced gene-gene edges (e.g., the cDCE respiration rate). Within a REFS^™^ network, conditional relationships represent either linearly-conditional or switched-conditional relationships (S1 Table). Therefore, instead of being represented by a node in the network, the discrete experimental conditions (censoring switches, the type of electron acceptor, and the type of electron donor) are used to modify the type of edge between two nodes. For length considerations, Table 1 displays conditional edges in the D2 consensus network only for those relationships with f > 0.85. The full list of all conditional edges in the D2 consensus network (both positive and negative correlated relationships) is presented in S2 Table.

**Table 1:** Gene-gene edges modified by a discrete variable in the D2 consensus network with high frequencies (f > 0.85).

f	r	Node 1	Description	Node 2	Description
If DCE (and all higher chlorinated ethenes, when provided) was available to be and was respired (1) or not (0)					
0.85	0.73	DET0006	histidyl-tRNA synthetase	DET1386	hypothetical protein
0.91	0.78	-	EA feed rate over the final 48 hrs	-	respiration rate of DCE
0.88	-0.23	-	respiration rate of PCE	DET0315	sensory box sensor histidine kinase
0.89	0.92	DET0564	ATP synthase subunit B	DET1263	DAK2 domain protein
0.89	0.92	DET1225	hypothetical protein	DET1417	formyltransferase
If TCE (and PCE, when provided) was available to be and was respired (1) or not (0)					
0.96	0.88	DET1477	hypothetical protein	DET1554	hypothetical protein
0.90	0.95	DET1510	hypothetical protein	DET0657	phosphoribosyltransferase
If PCE was available to be and was respired (1) or not (0)					
0.98	0.19	tRNA	Cys-1	tRNA	Gly-3
0.86	0.66	DET0414	hypothetical protein	DET0165	ISDet2, transposase orfA
0.90	0.93	DET1041	PQQ enzyme repeat domain protein	DET1039	SpoIIIJ-associated protein Jag
If DCE was detected above background levels (1) or not (0)					
0.94	0.32	DET0057	ATP-dependent Clp protease, ClpC	-	concentration of DCE
0.94	-0.72	DET0128	cobyrinic acid a,c-diamide synthase	DET0299	transcriptional regulator, Crp/Fnr family
0.85	0.81	DET0840	adenylosuccinate lyase	DET0439	FtsK/SpoIIIE family protein
0.87	-0.68	DET1430	HypA hydrogenase nickel insertion protein	-	methanogenesis rate
0.79	0.88	DET1431	HypB hydrogenase accessory protein	DET1433	hydrogenase assembly chaperone
0.94	0.74	DET1476	DNA binding domain, excisionase family	DET0981	tRNA pseudouridine synthase B
If PCE was detected above background levels (1) or not (0)					
0.88	0.84	DET1345	tagatose 1,6-diphosphate aldolase	DET1530	sensor histidine kinase
If an electron donor was being provided to the culture (1) or not (0)					
0.93	0.74	DET1084	DNA packaging protein, putative	DET1070	endolysin, putative
0.88	0.82	DET1244	NADPH-dependent FMN reductase	DET1005	transcriptional regulator, ArsR family
0.90	0.78	DET1570	hydrogenase, group 4, HycG subunit	DET1106	hypothetical protein
If the culture displayed inhibited growth (1) or not (0)					
0.86	0.90	DET0111	[Ni/Fe] hydrogenase, small subunit	DET0112	[Ni/Fe] hydrogenase, Fe-S cluster
0.86	0.74	DET0451	malate dehydrogenase, NAD-dependent	DET0115	ABC transporter, permease protein
0.92	0.81	DET0610	hypothetical protein	tRNA	Cys-1
0.99	0.76	DET1111	ATP-binding protein	DET0097	iron dependent repressor, putative
If the culture displayed inhibited growth because of DCE (1) or not (0)					
0.96	0.92	tRNA	Val-2	tRNA	Ser-1
0.99	0.88	DET0343	cell division protein FtsZ	DET0342	cell division protein FtsA
0.94	0.70	DET0383	hypothetical protein	DET1438	hypothetical protein
0.95	0.90	DET0591	hypothetical protein	DET0334	polyA polymerase family protein
0.85	0.77	DET0839	phosphoribosylaminoimidazole carboxylase	DET0607	hypothetical protein
0.87	0.85	DET0920	iron-sulfur cluster-binding protein	DET0921	hypothetical protein
0.92	0.86	DET1207	protein export protein, putative	DET0543	fatty acid/phospholipid synthesis
0.96	0.80	DET1350	DNA-binding response regulator	DET0922	twitching mobility protein
0.89	0.32	DET1446	excisionase family DNA binding protein	DET0432	DNA-binding response regulation

The relationship identified occurs when the discrete variable is set to 1. Gray squares indicate non-gene variables related to experimental conditions. The correlation score (r value) between the intensity values of two probes across all datasets is presented to display whether the relationship between the intensity values of the probes is positive or negative.

Notable conditional/switched relationships (Table 1) include the putative transcriptional regulator of the protein that presumably regulates the PceA reductive dehalogenase (the sensory box histidine kinase DET0315) displaying a strong and inverse relationship to the PCE respiration rate when chloroethene respiration is occurring (DCE is being respired; the censoring switch is set to 1). Histidine kinases (HK) often work in concert with response regulators (RR) in two-component transcriptional regulation (HK-RR). Because DCE is being respired when PCE, TCE, or DCE is fed, this condition essentially identifies chloroethene respiration but excludes datasets in which the culture is fed chlorophenols or no electron acceptor is provided. Therefore, this relationship indicates that an inverse correlation is highlighted for the DET0315 transcript levels and the respiration rate when *Dhc* strain 195 respires chloroethenes. This relationship suggests that the regulator acts as a potential repressor for the expression of *pceA*. Wagner et al. [35] suggested that *Dhc* RDase genes are likely regulated by a transcriptional repression process via a different type of regulator, MarR. Alternatively, DET0315 regulator is potentially controlling other processes that are responding inversely to the respiration rate.

By investigating the full list of positive conditional relationships between nodes (i.e., f ≥ 0.6; S2 Table), multiple relationships noted in a previous analysis of the D2 data using the SPINE approach [20] that link specified transcripts with inhibition were preserved in this REFS^™^ analysis. The main transcripts that were linked to the inhibited state in the previous SPINE analysis included DET0097 (a putative iron-dependent transcriptional regulator), DET0137 (a methylglyoxyl synthase), and DET0588 (a hypothetical protein), with REFS^™^ network edges that were driven by the inhibited condition strengths of 0.990, 0.000, and 0.812, respectively (Table 1 and S2 Table). The absence of DET0137 being conditional on inhibition highlights one of the drawbacks of the acyclic graph constraint applied in the REFS^™^ analysis (i.e., when A is linked to B and B to C, A cannot be linked to C). When more than two of the considered variables are behaving in a highly correlated manner, they potentially confound the selection of relationships. In this model, DET0137 is shared across two non-conditional network edges with DET0588 and the associated operon member DET0587 (f = 0.609 and 0.216, a total of 0.825; S3 File). Additionally, DET0588 is directly linked to DET0587 (f = 0.782, S1 File). Therefore, these set of genes are tightly linked, confounding the acyclic structure. However, the selection of DET0588 as conditional on inhibition reinforces the previous findings.

Two transcripts in the operon for the major periplasmic facing hydrogenase (Hup; DET0111 and DET0112) displayed a strong relationship with regards to the solvent toxicity/inhibition condition (f = 0.86). Both of these transcripts are down-regulated when the culture is inhibited by high chloroethene concentrations. Therefore, this relationship is likely displaying a switched type of response. When the culture is experiencing an inhibited state, the *hup* operon is substantially down-regulated. This relationship between the expression of the *hup* operon and *Dhc* respiration has been previously noted [29, 34], and *Dhc* ceased respiring when PCE reached saturating levels.

In contrast to the consensus network for D2, the KB-1^®^ consensus network displayed few significant edges modified by discrete variables. Only three relationships passed the significance cutoff (f ≥ 0.6): VS997 hypothetical protein/VS987 GTP cyclohydrolase were correlated only when the culture was fed in a batch rather than continuous manner (f = 1); VS1056 hypothetical protein/VS1054 transcriptional regulator when DCE is present (f = 1); and VS671 hypothetical protein/VS666 peptide deformylase when VC is present (f = 0.97). Because of the limited annotation of these transcripts, no conclusions can be drawn from these relationships, but notably, these pairs of transcripts are in close proximity on the *Dhc* strain VS genome.

### RDase gene networks in *Dhc* strains

**Fig 4:**
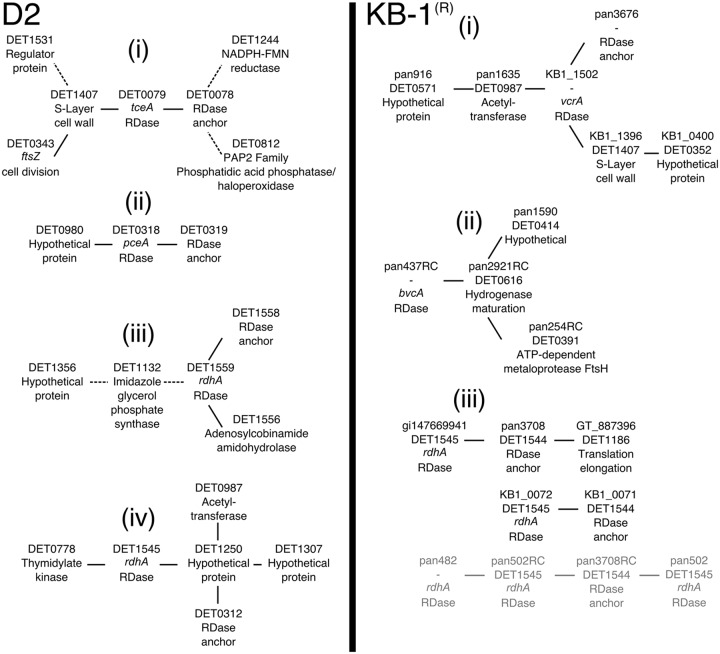
Transcripts in the consensus networks that are a maximum of two edges away from connecting with reductive dehalogenases in D2 (left) and KB-1^®^ (right). The four highest transcribed RDases in the D2 culture and the top five transcribed RDases in the KB-1^®^ culture are displayed. Other RDases are present in the final consensus network as well. The dashed lines are indicative of negative relationships, and the solid lines represent positive relationships. For the D2 consensus network, the transcript ID and a brief description is provided. For the KB-1^®^ consensus network, the probe ID, orthologous transcript ID in *Dhc* strain 195 (where applicable), and a brief annotation are provided. The grayed text in the KB-1^®^ culture represents transcripts from a minor Cornell-type strain.

The consensus network components for the nearest neighbors (within two edges) of the primary RDases in the D2 and KB-1^®^ cultures shows that the majority of RDases shown share an edge with their associated RDase anchoring protein (seven out of nine; Fig 4). A surprising strong positive linkage is noted between transcripts for the major RDase (TceA in the D2 culture and VcrA in the KB-1^®^ culture) with the transcript encoding for a putative S-layer cell wall protein (DET1407 in the D2 culture and KB1_1396 in the KB-1^®^ culture). A previous microarray study also displayed a tight correlation (R^2^ = 0.997) between the S-layer and *tceA* transcripts in a batch culture study of D2 [13]. This conserved relationship is notable because the S-Layer and major RDase proteins are among the most abundant proteins detected in proteomic assays for *Dhc* samples across cultures [17, 36–38]. As the major components of the cell wall and membrane-associated respiration processes, the expression of these transcripts may be tightly linked in both cultures because of their requirement for biomass growth. This hypothesis is supported by the *ftsZ* (DET0343) cell division transcript forming a strong edge (f = 0.966) to the S-layer transcript in the D2 consensus network (Fig 4). In addition to the *ftsZ*, a transcript for a RDase regulator, DET1531, is significantly, although inversely, connected to the expression of the transcripts encoding for the S-layer and TceA protein. Therefore, this regulator may be involved in the regulation of *tceA* or is responding in an inverse manner as *tceA* to the same environmental conditions. Notably, riboswitches appear upstream of both the coding region for the *tceA* and the S-layer genes, suggesting a potentially shared regulatory mechanism [39]. DET1531 should be explored in future studies as a potential additional regulatory element for the *tceA* and S-layer genes.

Four probes targeting orthologs of the DET1545 RdhA (an RDase with an unknown substrate range) were retained in the dataset after filtering for fluorescence intensity thresholds and correlations among ortholog probes: KB1_0072, gi147669941, panDhc_502, and panDhc_502_RC. Orthologs of DET1545 occur in almost all *Dhc* genomes currently available, and the dominant *Dhc* population in KB-1^®^ is known to encode an ortholog most closely related to the Cornell-type [7]. Based on percent probe identity patterns, the KB1_0072 probe targets Cornell-type DET1545 orthologs, gi147669941 targets Victoria lineage orthologs, and panDhc_502 and panDhc_502_RC target the Pinellas lineage (Fig 2). “Cross-talk” is limited by the number of mismatches with non-target sequences (90% identity for a 60-mer probe equates to six mismatches). The probes displayed the 29^th^ (KB1_0072), 55^th^ (gi147669941), 140^th^ (panDhc_502), and 470^th^ (panDhc_502_RC) highest maximum values among the 7,506 total probes on the pangenome array. In the correlograms (Fig 2) and REFS consensus network constructed from expression patterns (Fig 4), three distinct clusters for these DET1545 orthologs in the KB-1^®^ community can be detected. Within each of these clusters, an associated transcript encoding a RDase anchoring protein is directly connected to the DET1545 ortholog. The recruiting of distinct RDase anchoring subunits in the three clusters supports the hypothesis that three distinct DET1545-type transcripts are being captured and represented by the array.

This result highlights the fact that the pangenome array was able to detect simultaneous expression of homologous but non-identical genes across strains (black text (Cornell and Victoria type) versus gray text (Pinellas type) in Fig 4). However, this analysis was unable to connect these RDases to many other transcripts likely because of the high number of transcripts considered and lower number of experiments. Overall, methods like the pangenome array that resolve signals for DET1545 orthologs can be used to resolve distinguishable multiple strains of *Dhc* in the complex organohalide-respiring community KB-1^®^ and other communities in which the sequence diversity of DET1545 orthologs are known.

### Edges involving membrane bound oxidoreductases of the *hup* and *fdh*-like operons

**Fig 5:**
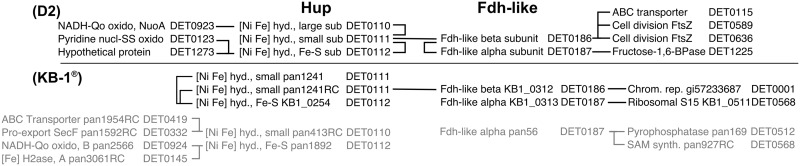
REFS^™^ consensus network summary for the *hup* and *fdh* transcripts. (a) D2 and (b) KB-1^®^. The connecting lines indicate edge strength scores that exceeded 0.6. Gray text in the KB-1^®^ culture indicates the minor stain of the Cornell/Victoria type. All relationships identified in the model between these transcripts were positive.

In the D2 culture, previous studies have shown a strong relationship in the expression pattern between two of the putative respiration associated oxidoreductases: the main uptake [Ni Fe] hydrogenase Hup (DET0110-0112) and the Fdh-like oxidoreductase (DET0185-0187) [26, 34]. Within the REFS^™^ consensus networks, these two operons are directly linked (through DET0111 and DET0186 in D2 and their respective homologs in KB-1^®^ for the major Pinellas-type strain (Fig 5)). In the D2 network, other transcripts that connect directly to these enzymes include those encoding for the NADH-ubiquinone oxidoreductase (DET0923), cell division proteins FtsZ (DET0589, DET0636), and the fructose 1,6-bisphosphatase; these transcripts link the *hup* and *fdh*-like transcripts to a broader network of transcripts encoding for oxidoreductases potentially involved in respiration and enzymes involved in cellular growth and division (see S3 File).

In the KB-1^®^ network, the major strain *fdh*-like transcript (alpha subunit) connects directly with a DET0001 chromosomal replicator homolog, suggesting that the *fdh*-like transcript is responding to growth-supporting environmental conditions. Additionally, a minor strain was detected with the pangenome probes; the encoded *hup* on the minor strain was linked to other oxidoreductases that were previously suggested to be involved in the respiration pathway of *Dhc* such as transcripts encoding for a NADH-ubiquinone oxidoreductase, Nuo (pan2566, homologous to DET0924), and a [Fe] hydrogenase, Hym (pan3061RC, homologous to DET0145) [26].

Combined, the results of the D2 and KB-1^®^ networks extend and broaden the previous findings for *Dhc* gene expression trends to two mixed communities, generalizing the previously identified relationship between the *hup* and *fdh*-like transcripts to multiple strains. The tight linkage between the *hup* and *fdh*-like transcripts in the KB-1^®^ network also agrees with previous findings [26], suggesting that the final Fdh-like enzyme may recruit a subunit from the expressed Hup operon to compensate for the missing FdhB subunit on all known *Dhc* genomes to date. Interacting proteins involved in respiration, including Hup and the Fdh with unknown function, have also been recently characterized at a proteomic level [40], highlighting the involvement of multiple operons in a single complex.

### Differentiation between major and minor strains in KB-1^®^

The ability to distinguish between the major and minor strains within the KB-1^®^ network analysis was seen across a wide range of genes. Of the 7,506 probes considered on the pangenome array, a total of 6,325 probes can differentiate between strains originating from the Cornell/Victoria vs. Pinellas lineages according to a BLAST analysis (with 85% similarity cutoff). In total, 3,010 and 3,315 were predicted to determine orthologs from Cornell/Victoria and Pinellas strains, respectively. Within the reconstructed network for the KB-1^®^ community, a total of 722 significant edges are predicted to connect two nodes within a group (421 Pinellas-Pinellas and 301 Cornell/Victoria-Cornell/Victoria), whereas only 208 significant edges connect nodes from different *Dhc* groups (Pinellas-Cornell/Victoria). Therefore, strain specific nodes are more likely to connect with other nodes from the same cluster; the consensus network therefore represented at least two populations of *Dhc*. Overall, the slight genomic variations between strains of *Dhc* [41] lead to different survival patterns, metabolic capabilities, and environmental responses as experimentally displayed in the suitability of various strains to differing culturing conditions in Marshall et al. [42].

## Summary

This study applied the Bayesian REFS^™^ gene network inference platform to heterlogous datasets comprised of gene transcript levels and metabolite data for two organohalide-respiring communities containing *Dhc* (D2 and KB-1^®^). For the D2 community, the developed consensus network highlighted relationships between genes of interest and experimental conditions such as the discussed potential *pceA* regulator being inversely related to the PCE respiration rate. For the KB-1^®^ community, the pangenome array that was utilized in this study was found to be capable of capturing distinct signals from multiple populations of *Dhc* in KB-1^®^; this strain distinction was further captured in the inferred consensus network. Therefore, the pangenome microarray provides a good alternative to sequencing based transcriptomic methods to capture multiple strains. Comparing the consensus networks for D2 and KB-1^®^ revealed a strong conserved relationship between the major RDase (TceA or VcrA) and the putative S-layer cell wall protein. Future experiments should investigate the mechanism of this relationship by exploring the upstream promoter regions of these genes or performing heterologous co-expression analyses. Additionally, the relationship between transcripts encoding for the Hup and Fdh-like complexes was noted in both the D2 and KB-1^®^ consensus networks, suggesting a generalized pattern across *Dhc* strains from the Cornell/Victoria and Pinellas groups. From these findings, the REFS^™^ gene network inference platform produced both predictive and confirmatory relationships, displaying the utility of this method.

To extend this study and provide further insights into functionally unknown proteins, additional perturbation experiments should be performed. These perturbations should focus on pathways of interest (e.g., cobalamin incorporation, stress responses) and on providing an extended portfolio of electron acceptors other than chlorinated ethenes and chlorinated phenols (e.g., chlorobenzenes, brominated compounds). The additional electron acceptors could assist in elucidating the roles of functionally unknown RDases. With the recent publication of a heterologous expression method for RDases, these functional predictions can then be confirmed at a proteomic level [10]. From this current study, a homolog of DET1545 is a good candidate to investigate with this method because of its high transcript expression and being conserved across multiple *Dhc* strains.

## Supporting Information

S1 Fig Chloroethene concentration versus time for the KB-1^®^ batch culture time-series. Dechlorination profiles showing the total amount of chloroethenes (TCE, DCE, VC) and ethene (ETH) detected normalized to liquid culture volume for the KB-1^®^ cultures batch fed 220 microM TCE for the entire experiment. Data labels indicate the specific metabolites. Samples (50 mL duplicate cultures) were sacrificed for RNA analysis at 4.2, 8.3, 13.7, 23.1, 27.9, and 69.7 hours post batch feeding of TCE.
(TIFF)

S2 Fig Chloroethene concentration versus time for the KB-1^®^ batch trichloroethane-stress series. Dechlorination profiles showing the total amount of chloroethenes (TCE, DCE, VC) and ethene (ETH) normalized to liquid culture volume for the KB-1^®^ cultures batch fed 220 microM TCE for the entire experiment. Experiment titles for the samples indicate the time the cultures were sacrificed after the TCA stressor was added. Cultures sampled at 24 hours and 40 hours as a control (a,b); 17 hours post trichloroethane (TCA) amendment (c,d), and 48 hours post TCA addition (e,f). Data labels indicate the specific metabolites. The red bar denotes the time of 22 microM TCA addition (added to the stress cultures after 20 hours from start of experiment).
(TIFF)

S1 Table Continuous and discrete experimental variable considered in the REFS^™^ network analysis for the D2 culture. (XLSX)

S2 Table All conditional edges appearing in the D2 consensus network. (XLSX)

S1 File The data table used to construct the D2 consensus network. (TXT)

S2 File The data table used to construct the KB-1^®^ consensus network. (TXT)

S3 File D2 consensus network xgmml file. (XGMML)

S4 File KB-1^®^ consensus network xgmml file. (XGMML)
