# Supplementary figures and images for: Inferring Gene Networks for Strains of Dehalococcoides Highlights Conserved Relationships between Genes Encoding Core Catabolic and Cell-Wall Structural Proteins
Source: PLoS One. 2016 Nov 9;11(11):e0166234. doi: 10.1371/journal.pone.0166234 (PMC5102406; doi:10.1371/journal.pone.0166234)

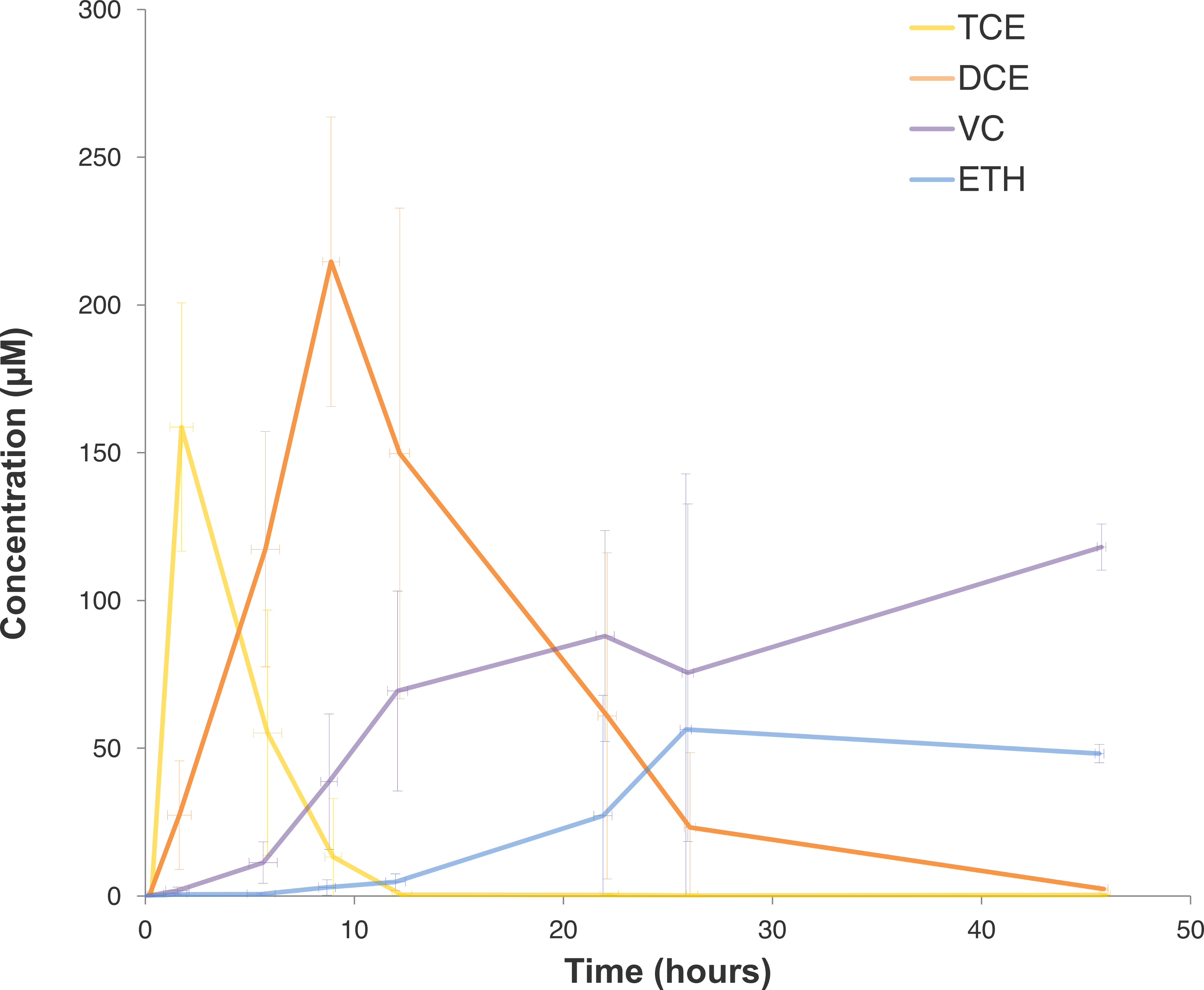

Supplement: S1 Fig — Dechlorination profiles showing the total amount of chloroethenes (TCE, DCE, VC) and ethene (ETH) detected normalized to liquid culture volume for the KB-1® cultures batch fed 220 microM TCE for the entire experiment. Data labels indicate the specific metabolites. Samples (50 mL duplicate cultures) were sacrificed for RNA analysis at 4.2, 8.3, 13.7, 23.1, 27.9, and 69.7 hours post batch feeding of TCE. (TIFF) [file pone.0166234.s001.tiff]

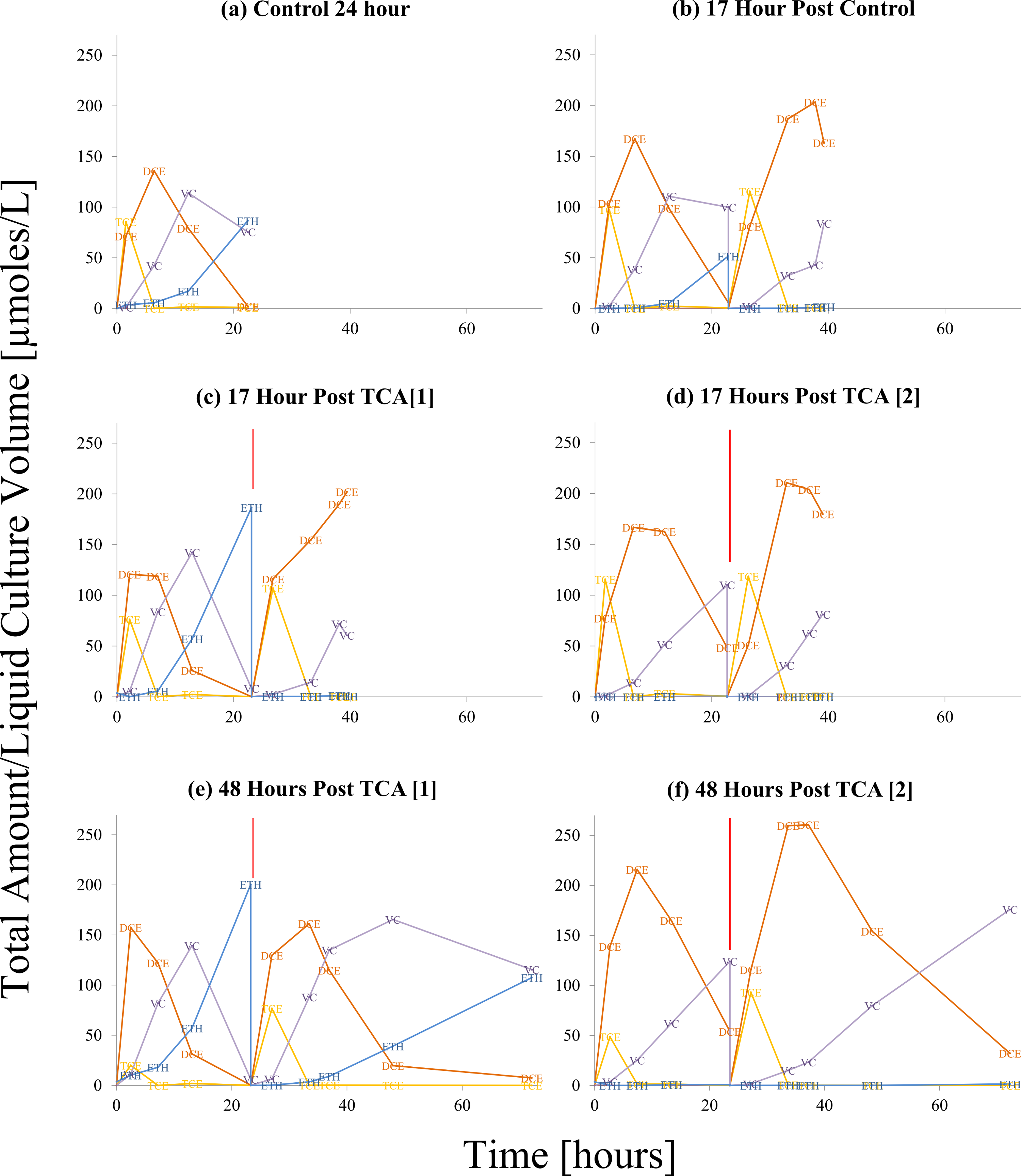

Supplement: S2 Fig — Dechlorination profiles showing the total amount of chloroethenes (TCE, DCE, VC) and ethene (ETH) normalized to liquid culture volume for the KB-1® cultures batch fed 220 microM TCE for the entire experiment. Experiment titles for the samples indicate the time the cultures were sacrificed after the TCA stressor was added. Cultures sampled at 24 hours and 40 hours as a control (a,b); 17 hours post trichloroethane (TCA) amendment (c,d), and 48 hours post TCA addition (e,f). Data labels indicate the specific metabolites. The red bar denotes the time of 22 microM TCA addition (added to the stress cultures after 20 hours from start of experiment). (TIFF) [file pone.0166234.s002.tiff]
